# Rare fibrolipoma of the tongue: a case report

**DOI:** 10.1186/s13256-015-0653-1

**Published:** 2015-08-21

**Authors:** Giorgio Iaconetta, Marco Friscia, Atirge Cecere, Antonio Romano, Giovanni Dell’Aversana Orabona, Luigi Califano

**Affiliations:** 1 Department of Neurosurgery, University of Salerno, Via Giovanni Paolo II, 132, 84084 Fisciano, SA Italy; 2 Department of Maxillofacial Surgery, University of Naples Federico II, Via Sergio Pansini, 5, 80133 Naples, Italy

**Keywords:** Fibrolipoma, Tongue, Lipoma, Infiltrating

## Abstract

**Introduction:**

Lipoma is a benign tumor infrequent in the oral cavity, particularly in the tongue: indeed, lipomas only represent approximately 0.3% of all tongue neoplasia. Compared to conventional lipoma, fibrolipoma of the tongue is a very rare lesion that accounts for around 25–40% of tongue lipomas, and until now, to the best of our knowledge, only 14 cases have been described in which histological diagnosis of fibrolipoma was specifically confirmed. We report the case of a patient with a voluminous fibrolipoma of the tongue, treated by means of surgical excision. Fibrolipoma excision, like that described in this report, sometimes may be laborious, because fibrous bands appear to be focally infiltrating adjacent tissues, giving rise to some doubts about the nature of the lesion.

**Case presentation:**

We report the case of a voluminous fibrolipoma of the tongue in a 71-year-old Caucasian woman.

**Conclusions:**

Because of its histological characteristics, abundance of connective and secondary changes/atrophy, fibrolipoma may appear as infiltrating adjacent tissues and may cause doubts of differential diagnosis with malignant infiltrating lesions. Surgical excision is the elective treatment. However, an accurate differential diagnosis, postsurgical histological examination and careful follow-up are required.

## Introduction

Lipoma is a benign tumor most frequently found in almost all anatomical sites that have adipose tissue in their structure.

However, its presence is relatively infrequent in the oral cavity. The tongue in particular, totally lacking in adipose tissue, is a rare location for a lipoma; indeed lipomas only represent approximately 0.3% of all tongue neoplasia. According to a histological criterion, the World Health Organization (WHO) [1] classifies lipomas in: conventional lipomas, fibrolipomas, angiolipomas, pleomorphic lipomas/spindle cell, mixolipomas, condrolipomas, osteolipomas, miolipomas, lipomatosis, lipomatosis of the nerve, lipoblastomas, and hybernomas. Compared to conventional lipoma, other histological variants are much more rare. Particularly rare is the fibrolipoma that, sometimes, but for its histological characteristics, may cause doubts of differential diagnosis with infiltrating lesions. Until now, to the best of our knowledge, 185 cases of tongue lipoma have been described and, among these, only 14 cases [2–10] have been described in which a histological diagnosis of fibrolipoma has been specifically confirmed (see Table 1). This estimate is derived from a count of all the cases cited in the PubMed database found by entering the keywords ‘tongue’ and ‘lipoma’. We report the case of a patient with a voluminous fibrolipoma of the tongue, treated by means of surgical excision.

**Table 1:** Summary of previous reported cases of tongue fibrolipoma

Author	Number of cases
Horton et al. 1968 [5]	1
Dattilo et al. 1996 [3]	1
Epivatianos et al. 2000 in Manjunatha et al. [2]	2
Said-Al-Naief et al. 2001 [6]	3
Fregnani et al. 2003 [7]	1
Juliasse et al. 2010 [9]	1
Manor et al. 2011 [10]	3
Shi et al. 2014 [4]	1
Camacho et al. 2014 [8]	1

## Case presentation

**Fig. 1:**
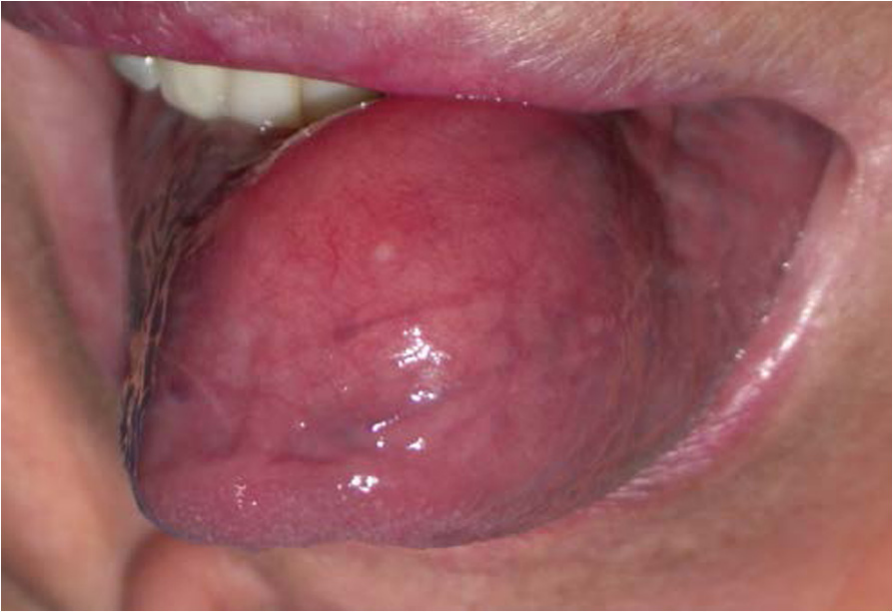
Tongue fibrolipoma. Our patient presented with a swelling of the anterior two-thirds of the ventral surface of her tongue

A 71-year-old Caucasian woman presented to our hospital with a painless swelling on the ventral surface of her tongue (Fig. 1). For the previous 30 days, she had had dysfunction of phonation and swallowing, and a sensation of ‘obstruction’ of the oral cavity.

Our patient did not manifest other pathologies except for moderate hypercolesterolemia. The neoformation, involving the anterior portion of the ventral surface of her tongue, had a 40mm maximum diameter, a curvy shape and soft consistency, was movable on the superficial and deep plans and, furthermore, was covered by mucosa, which appeared to be normal in color and trophism. After a clinical evaluation, an in-depth diagnostic analysis using computed tomography (CT) was undertaken.

**Fig. 2:**
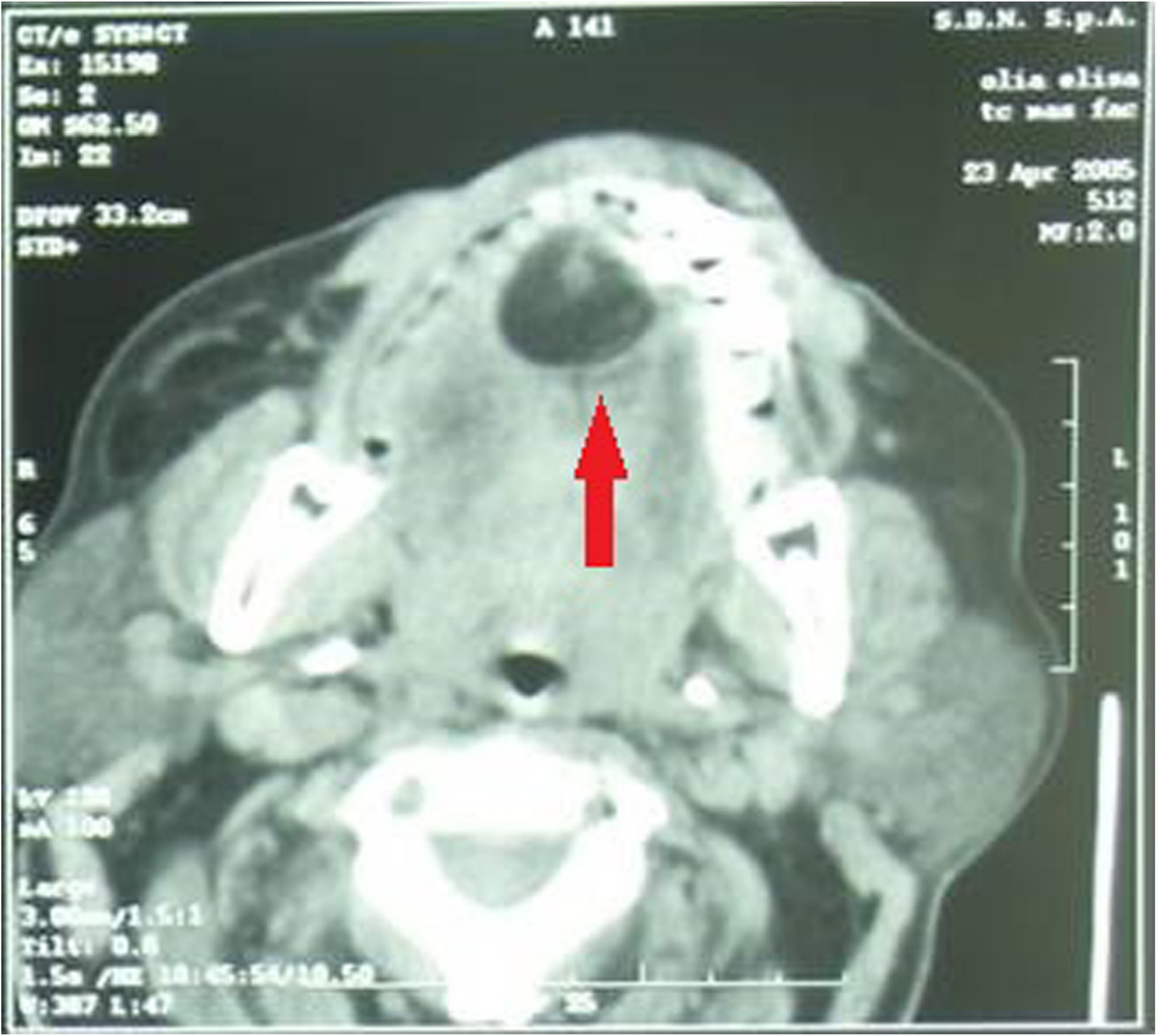
Computed tomography image of the fibrolipoma. A computed tomography scan (after contrast administration) shows an ovalar-shaped mass with distinct margins. The inhomogeneous density of the mass is due to the presence of solid spots in a fluid content

A contrast-enhanced CT scan showed an oval-shaped neoplasm, with distinct margins, and a rather patchy density (Fig. 2). Nonetheless, its features seemed to confirm the adipose composition of the mass. Therefore, after clinical and instrumental evaluation, surgical treatment was suggested. Under local anesthesia, an intraoral approach was planned in order to perform the removal of the mass, by means of a vertical mucosal incision on the midline, on the inferior surface of the tongue. Intraoperatively, the excision was more laborious at some points because fibrous bands appeared to be focally infiltrating surrounding tissues, and the neoformation was hard to detach (giving rise to some doubts about the nature of the lesion).

**Fig. 3:**
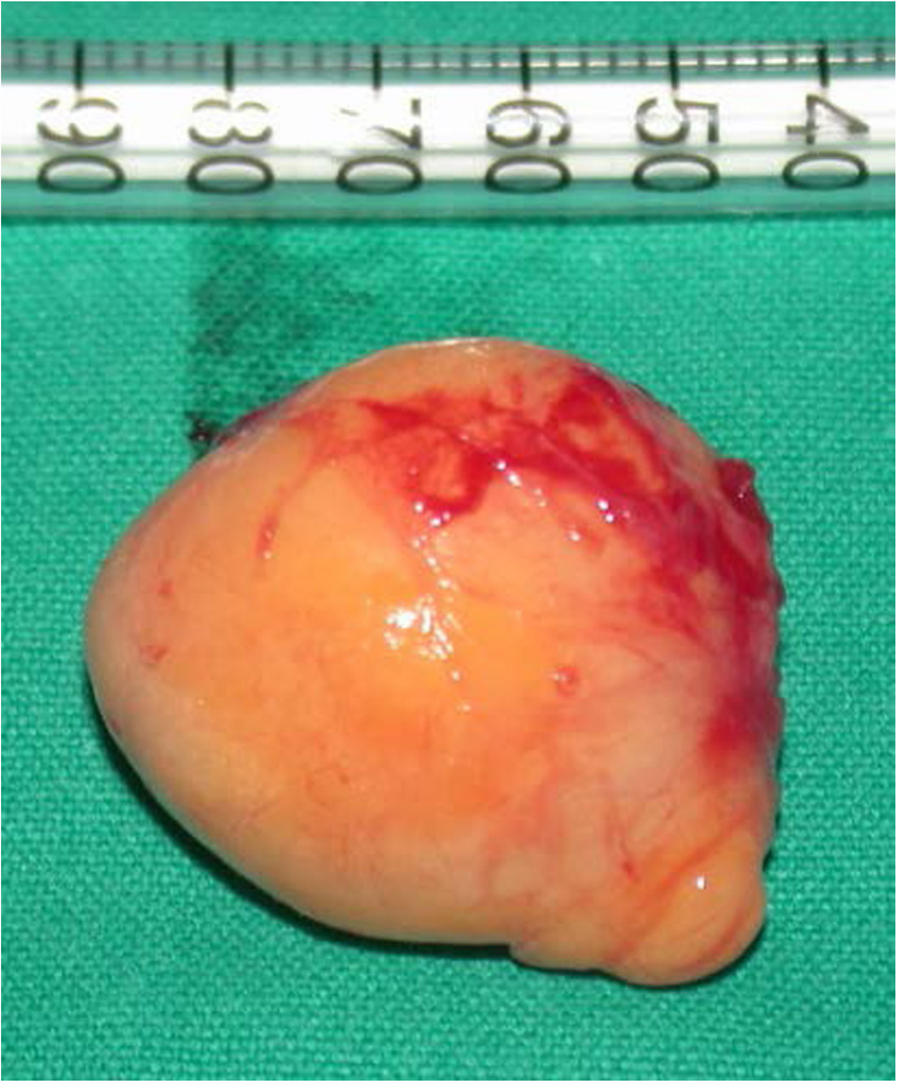
Enucleated fibrolipoma. On macroscopic examination, the neoplasm appears capsulated, yellowish and soft in consistency, and 40 × 40mm in size

On macroscopic examination (Fig. 3), the mass appeared to be capsulated, soft and yellowish in color, and 40 × 40mm in size.

**Fig. 4:**
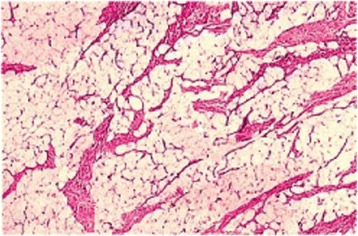
Histological features. Hematoxylin and eosin stain: mature fat tissue, sepimented by fibrous strands, clusters of unvacuolated fat cells forming lace-like sheets, bland peripheral nuclei and mild nuclear atypia are shown

On histological examination, the lesion showed clear characteristics of mature adipose tissue, without atypical aspects, subdivided by fibrous shoots in multiple adipose vacuums (Fig. 4); peripherally, fibrous connective tissue was well represented and the mass was rolled up in a connective fibrous tissue capsule, on which it was possible to observe numerous fibrous bands; hence the histological evaluation classified the lesion as a fibrolipoma.

The postoperative period was uneventful and our patient was discharged a few days later, with her tongue functions perfectly safeguarded.

## Discussion

Lipomas are the most common benign neoformation in almost all anatomical sites that present adipose tissue in their structure. They represent 13–20% [2] of head and neck tumors, and 1–5% of neoplasms of the oral cavity [1–3, 11].

In particular, tongue lipomas are extremely rare and represent 0.3% of all neoplasms of the tongue [12].

On the basis of the results of research conducted on the PubMed database (performed by searching for the keywords ‘tongue’ and ‘lipoma’), since 1912, 185 cases of lipoma of the tongue have been reported and 14 cases of tongue fibrolipoma [2–10]. Tongue lipomas are more frequent during the fourth to the sixth decade. As reported by some authors [1], a male predilection was noted from the literature, in contrast, others have also shown a female prevalence. Lingual lipomas can be single or multiple, and may be either isolated or inscribed as part of syndromes such as Gardner syndrome, Bourneuille syndrome, Gorlin syndrome, and various syndromes characterized by macroglossia.

They are generally localized under the mucous membrane. However, Colella et al. [1] have described cases of intramuscular lipomas of the tongue.

According to a histological criterion, the WHO [1] classifies lipomas in: conventional lipomas, fibrolipomas, angiolipomas, pleomorphic lipomas/spindle cell, mixolipomas, condrolipomas, osteolipomas, miolipomas, lipomatosis, lipomatosis of the nerve, lipoblastomas, and hybernomas.

Compared to conventional lipoma, other histological variants are much more rare, and fibrolipoma is particularly rare [1]. Until now, specifically, only 14 cases have been described in which histological diagnosis of fibrolipoma was confirmed [2–10].

They vary in dimensions, and lipomas of considerable size have been described [1, 3]. The most common site for fibrolipomas is the buccal mucosa, followed by the tongue [2]. Sessile and pedunculated fibrolipomas have been described [2].

Histologically, fibrolipoma is composed of mature fat cells subdivided into lobules by fibrous shoots. Fibrous connective tissue, especially peripherally, is well represented. From a connective fibrous capsule originate multiple fibrous bands, that often adhere tenaciously to adjacent tissues and structures, with focally pseudo-infiltrating aspects that may cause doubt of a differential diagnosis with malignant infiltrating lesions.

It generally presents an ovalar shape with variable volume and dimensions, with a yellow color, and the consistency is generally soft or semi-firm [1]. When compared to conventional lipoma, even macroscopically, the fibrous component appears more represented, especially in the capsule. It is typically a slow-growing tumor.

The etiopathogenesis of lipomas and fibrolipomas is still unknown, even though an alteration of the lipidic metabolism or an anomalous localization of fatty-fetal tissue in the tongue have been suggested. It is thought that repeated mild trauma may trigger fatty tissue proliferation [11], as suggested by Kiehl et al. [13], who described a fibrolipoma beneath a complete mandibular denture.

Diagnosis is made by an accurate anamnesis, which can reveal a relation with lipidic metabolism alterations, and a good clinical examination.

Clinically, tongue fibrolipomas are generally asymptomatic, not painful, although, because of the space they occupy, they can cause chewing, swallowing or phonation dysfunction. As noted by Manjunatha et al. [2], it is interesting to observe how clinically, several cases of fibrolipoma are diagnosed as ‘fibroma’, because of their semi-firm consistency (which is different from the soft consistency of conventional lipomas).

Adding to the clinical data or when differential diagnosis is more complex, imaging techniques can also be used, such as a CT scan, together with diagnostic fine needle aspiration cytology (FNAC), which can be helpful to discriminate the nature of the mass. The CT features of fibrolipoma show an ovalar mass with definite margins, and inhomogeneous density (compatible with adipose tissue). Although cytology can guide us, in many cases it is not sufficient to confirm the diagnosis of fibrolipoma absolutely.

In fact, because of its pseudo-infiltrating aspects and its tenacious adherence to surrounding structures, histological examination is mandatory to clarify the nature of the neoformation and resolve all doubts. For its histological characteristics, for its possible secondary changes [14], for its adhesion and focal pseudo-infiltration of surrounding tissues (due to the abundance of collagen and connective tissue), fibrolipoma sometimes may cause doubts of differential diagnosis with malignant infiltrating lesions [13, 15]. Cases of tumors of the tongue mistaken for other diseases have been reported in the literature. D’Antonio et al. described the case of a woman affected by a pleomorphic lipoma of the tongue simulating a liposarcoma [15]. Furthermore, as reported by some authors, fibrolipoma is characterized by a greater proliferative activity than other simple variants, therefore an accurate diagnosis and histological examination is mandatory [2].

Differential diagnosis has to be performed among different pathologies [1, 8, 13, 15], such as pyogenic granuloma, lymphangioma, schwannoma, dermoid cyst, ectopic thyroid tissue, minor salivary glands neoplasm, angiomas, and infiltrating tumors such as liposarcoma.

Treatment consists of surgical excision, according to the classical technique or by the use of diode laser [2].

## Conclusions

According to the literature, fibrolipoma of the tongue is a very rare lesion, which accounts for approximately 25–40% of tongue lipomas [7], and until now only 14 cases have been described with histological diagnosis of fibrolipoma specifically confirmed.

Due to the abundance of collagen, connective tissue, fibrous bands, possible ulcerative changes of the coating mucosa [3], and secondary changes/atrophy [4], fibrolipoma may appear as infiltrating adjacent tissues and may cause doubts for differential diagnosis with malignant infiltrating lesions, such as liposarcoma [15]. Surgical excision is the elective treatment. An accurate differential diagnosis, postsurgical histological examination and careful follow-up are, however, required.

## Consent

Written informed consent was obtained from the patient for publication of this case report and any accompanying images. A copy of the written consent is available for review by the Editor-in-Chief of this journal.
